# A semi-synthetic glycosaminoglycan analogue inhibits and reverses *Plasmodium falciparum* cytoadherence

**DOI:** 10.1371/journal.pone.0186276

**Published:** 2017-10-18

**Authors:** Mark A. Skidmore, Khairul Mohd Fadzli Mustaffa, Lynsay C. Cooper, Scott E. Guimond, Edwin A. Yates, Alister G. Craig

**Affiliations:** 1 School of Biological Sciences, University of Liverpool, Crown Street, Liverpool, United Kingdom; 2 Liverpool School of Tropical Medicine, Pembroke Place, Liverpool, United Kingdom; 3 School of Life Sciences, Keele University, Huxley Building, Keele, Staffordshire, United Kingdom; Institut national de la santé et de la recherche médicale - Institut Cochin, FRANCE

## Abstract

A feature of mature *Plasmodium falciparum* parasitized red blood cells is their ability to bind surface molecules of the microvascular endothelium via the parasite-derived surface protein *Plasmodium falciparum* erythrocyte membrane protein 1 (PfEMP1). This ligand is associated with the cytoadherence pathology observed in severe malaria. As pRBC treated with effective anti-malarial drugs are still able to cytoadhere, there is therefore a need to find an adjunct treatment that can inhibit and reverse the adhesion process. One semi-synthetic, sulfated polysaccharide has been identified that is capable of inhibiting and reversing sequestration of pRBC on endothelial cells *in vitro* under physiological flow conditions. Furthermore, it exhibits low toxicity in the intrinsic (APTT assay) and extrinsic (PT assay) clotting pathways, as well as exhibiting minimal effects on cell (HUVEC) viability (MTT proliferation assay). These findings suggest that carbohydrate-based anti-adhesive candidates may provide potential leads for therapeutics for severe malaria.

## Introduction

An important characteristic of the pathogenesis of severe malaria (SM) results from the ability of parasitized red blood cells (pRBC) to sequester in the microvasculature, supported by post-mortem studies of cerebral malaria (CM) that indicate high levels of pRBC bound in brain microvessels [1, 2]. The involvement of sequestration in pathogenesis could be a result of microvasculature occlusion, and/or downstream effects caused by interactions between pRBC and the endothelium, including local inflammatory responses. [3].

The cytoadherence of pRBC to vascular endothelial cells occurs when PfEMP1, a parasite derived molecule present on the surface of pRBC, binds to several distinct adhesion molecules present on the surface of host endothelium. Previous studies have shown that parasite isolates from children with SM bind to several receptors, suggesting that synergistic effects between adhesion molecules may contribute to malaria pathophysiology. Yipp and others indicated that, in some cases, multiple receptors may be involved in adhesion, and recent data suggest that ICAM-1 and EPCR binding play a role in cerebral malaria [4–6]. The role of cytoadherence in SM, coupled with splenic evasion, suggests that a compound capable of reversing this adhesive phenotype would be desirable in terms of reducing clinical disease. Previous work has concentrated on inhibiting cytoadherence, whereas for actual cases of malaria it will be important that inhibitors of adhesion should also be able to reverse existing adhesion.

It is preferable that antimalarial drug treatment is able to kill parasites in the non-adhesive, ring stages to help prevent the next wave of pRBC from sequestrating and thus artemisinin is a good choice. This might explain the reduced mortality observed in field studies from Thailand (SEQUAMAT) [7] and Africa (AQUAMAT) [8] in which artemisinin and quinine (which kills exclusively mature pRBC) were compared. Despite this, there remains a high mortality rate accounting for in excess of 50% of deaths during the first 48 hours following hospital admission that is largely unaffected by the use of artemisinin-based combination therapies (ACTs). This may be due to the pRBC already having been sequestered to the endothelium. Consequently, there is a need for adjunct therapies to support the critically ill patient, which can be used in combination with antimalarials such as artemisinin, to remove the sequestered pRBC mass or reduce its effects on the host, whilst conventional drugs kill the parasite effectively.

Polysaccharides, which are found throughout both the animal and plant kingdoms, serve diverse functions in their tissues of origin and are frequently complex and heterogenous in structure. In plants, they include acidic polysaccharides, usually as a result of the presence of carboxylate groups (e.g. alginates and pectins) or O-sulfates groups (carrageenans), some of which tend to form gels, often dependent on their association with divalent cation(s). However, it is also possible to introduce other acidic groups (such as O-sulfates) by chemical means and these modified plant polysaccharides exhibit a range of biological activities in mammalian systems that arise from their ability to mimic the binding properties of the mammalian glycosaminoglycan (GAG) class of extracellular polysaccharides [9], which interact with many proteins.

Modified, semi-synthetic polysaccharides are capable of binding distinct proteins with several levels of specificity. While highly acidic macromolecules could potentially interact in a non-specific and non-physiologically relevant manner with proteins, several highly negatively charged, sulfated polysaccharides, such as heparin (hep), heparan sulfate (HS), chondroitin sulfate (CS), dextran sulfate, fucoidan, as well as the non-sulfated glycosaminoglycan hyaluronic acid (HA), confer high affinity for particular proteins. Furthermore, anionic carbohydrates have been reported to inhibit erythrocyte invasion by *Plasmodium* merozoites, cytoadherence of pRBC to host cells, and to disrupt rosette formation between pRBC and uninfected erythrocytes [10].

Polysaccharides with different levels and patterns of sulfation have been demonstrated to inhibit *P*. *falciparum* growth and interfere with the adhesion of pRBC to the host endothelial receptor CD36 [11–13]. Chemically O-sulfated cellulose was able to inhibit adhesion to CSA expressed on both CHO cells and placental tissue [14]. Cellulose sulfate (*ca*. 50 kDa), with significant levels of O-sulfate substitution at positions-2 (44%) and -3 (36%) of the glucose repeating units, showed the most favourable inhibitory capacity and was able to reverse bound pRBC to CHO cells and placental tissue [14]. In addition to being directly involved in adhesion, it has been reported that sulfated CSA is also able to inhibit and reverse adhesion of CSA-adherent pRBC *in vitro* [14] and in splenectomised monkeys *in vivo* [15]. Furthermore, altering selected functional groups, especially sulfates on the saccharide branches, showed that it is possible to reduce binding, and in some cases augment the attachment of the pRBC to endothelium, all mediated by sulfated polysaccharides [16]. Other work using a modified derivative of heparin (sevuparin) has demonstrated the ability of modified glycosaminoglycans to inhibit cytoadherence and rosetting (the binding of infected erythrocytes to uninfected red blood cells) [17–19]).

In this study, a variety of semi-synthetic, chemically modified polysaccharides with various levels and patterns of sulfation, derived from important industrial polysaccharides, were tested for their capacity to inhibit and reverse malaria cytoadherence, which may contribute to the development of novel therapeutics capable of targeting adhesion of pRBC to receptors such as ICAM-1 and CD36 on endothelial cells.

## Materials & methods

### Endothelial cells

Primary human umbilical vein vascular endothelial cells (HUVEC) and human dermal microvascular endothelial cells (HDMEC) obtained from Promocell were cultured as per the manufacturer’s instructions. The Human Brain Endothelial Cell line (HBEC5i) was cultured as described by Tripathi *et al*. [5]. Primary endothelial cells at passage 4–6 were used for all experiments. Prior to use, cells were stimulated by addition of 1 ng.ml^-1^ TNF for 18 h to allow enhanced ICAM-1 expression on the surface of the endothelial cells.

### Parasite culture

ItG (ITvar16) [20] and A4 (ITvar14) [21] laboratory parasite lines, which are well characterized for their binding to ICAM-1 and CD36 [22], were cultured under standard conditions in RPMI 1640 medium supplemented with 37.5 mM HEPES, 7 mM D-Glucose, 25 ug.ml^-1^ gentamycin sulfate, 2 mM L-glutamine and 10% (v/v) pooled human serum at pH 7.2 in a gas mixture comprising 96% nitrogen, 3% carbon dioxide, and 1% oxygen. Additionally, recently culture adapted patient isolates: PO69, 8146 and 8026 were also included in the study [23].

### Plasmagel trophozoite enrichment

Parasite culture at trophozoite stage was centrifuged (500 g, 5 min) and the pRBC pellet was resuspended in a ratio of 2 volumes pellet to 3 volumes RPMI-based growth media without human serum (incomplete medium) and 5 volumes Plasmion (Laboratoire Fresenius Kabi, France), and allowed to settle for 20–30 min at 37 C. Trophozoite stage pRBC in the top layer were washed three times in incomplete medium and the parasitaemia assessed by Giemsa stained smear.

### Selection of pRBC on ICAM-1 purified protein

To increase the homogeneity of the ItG parasite population that expresses a PfEMP-1 protein with high affinity for ICAM-1, the population was subjected to selection on ICAM-1 protein. 2.5 μg of ICAM-1 protein was coated on to 50 μl of protein A Dynabeads (Invitrogen) in 200 μl of 1% (v/v) Bovine Serum Albumin (BSA) in PBS and incubated for 1 hour at room temperature with gentle rotation (15 rpm). Dynabeads were washed with 1% BSA/PBS and a magnetic stand. 50 μl of synchronized and enriched ItG parasite culture using Plasmion were incubated with the coated beads in 400 μl 1% BSA/PBS for 45 min at room temperature by gentle rotation. Bound pRBC were washed 3 times with 1% BSA/PBS using the magnetic stand. Beads were resuspended in 5 ml of complete RPMI media and transferred to culture in a T25 culture flask with the addition of 100 μl of washed red blood cells.

### Chemical sulfation of polysaccharides

Carbohydrate precursors were O-sulfated by a modified version of the chlorosulfonic acid (CSA) sulphation method as described previously [9, 24]. Briefly, precursor carbohydrates were dissolved in ice-cooled dimethylsulfoxide with pyridine, before chlorosulfonic acid was added dropwise. The mixtures were held at 95°C for 2 hr, cooled over ice and slowly neutralized with sodium hydroxide. Ethanol precipitations were performed prior to extensive dialyses (7 kDa cut-off membrane) against distilled water. Samples were lyophilized and stored at 4°C prior to use.

### Static inhibition adhesion assay screening of the chemically modified, semi-synthetic anionic polysaccharides on endothelial cells

Initially, solutions of 44 chemically-modified semi-synthetic anionic polysaccharides (see Supplementary Data) were diluted to 1 mg.ml^-1^ in binding buffer (RPMI 1640 with 25 mM HEPES, 11 mM D-Glucose, 2 mM L-glutamine pH 7.2) and screened for their anti-adhesive properties using a static cell binding based assay as described by [22]. HUVEC, (2-6^th^ passage) were seeded onto 1% w/v gelatin coated 13 mm Thermanox coverslips (Nunc). Once confluent, the cells were incubated overnight at 37°C with 1 ng.ml^-1^ TNF (Biosource International). Cells were then washed with binding buffer prior to use. A suspension of 3% pRBC and 1% HCT containing 1 mg.ml^-1^ of compound was allowed to bind, for 1 hour with mixing every 10 minutes, and following two dip washes, coverslips were placed in a gravity wash for 30 min. Coverslips were then transferred to a second gravity wash for 10 min, fixed in 1% v/v glutaraldehyde and stained with Giemsa. Control experiments lacking the addition of the test compounds were also included. Coverslips were dried and mounted on slides using DPX mountant (Sigma). Levels of adhesion were quantified by microscopy under 300x magnification. The number of adherent pRBC per mm^2^ was calculated.

### Flow adhesion assay inhibition by the chemically modified, semi-synthetic anionic polysaccharides on endothelial cells

This type of assay attempts to mimic the conditions seen in the post capillary venule by allowing pRBC to flow over slides coated with endothelial cells. Permanox chamber slides (Nunc) were coated with 1% (w/v) gelatin for 1 hr at 37°C, seeded with endothelial cells (HUVEC; HDMEC; HBEC5i) and incubated until confluent. Confluent slides were then incubated overnight at 37 C with 1 ng.ml^-1^ TNF prior to use. PRBC suspensions (3% parasitaemia and 1% HCT) with or without 1 mg.ml^-1^ modified polysaccharides were flowed over the endothelial cells for 5 min, followed by binding buffer (without the compounds) for 2 min to remove unbound cells. The flow rate yielded a wall shear stress of 0.05 Pa, used widely to mimic wall shear stresses in the microvasculature. The number of adherent pRBC was counted in six separate fields under 300x magnification and the density of parasitized red blood cells per unit area (pRBC.mm^-2^) was calculated. All assays were carried out at 37 C and were performed in duplicate or triplicate on three independent occasions.

### Flow adhesion assay reversal by the chemically modified, semi-synthetic anionic polysaccharides on endothelial cells

Reversal cell assays were carried out using a similar procedure to the flow inhibition adhesion cell assay but with an initial phase in the absence of compounds. PRBC at 3% parasitaemia and 1% HCT were flowed through the slide for 5 min to allow for pRBC adhesion. Flow was continuous throughout the experiment at 0.05 Pa shear stress. Binding buffer was used to remove unbound pRBC. Timing was started at the beginning of this wash, which continued for 2 min before the binding medium was swapped for medium containing the modified compound being tested. The number of bound cells in six fields along the slide was counted at 0, 5, 10, 15 and 20 min. The number of adherent cells counted was used to calculate the pRBC bound per mm^2^.

### Prothrombin time (PT) coagulation assay

Samples, controls and Thromborel S reagent (Siemens) were pre-warmed to 37 C prior to use. Serially diluted, sulphated carbohydrate samples (50 μl) were incubated with normal human citrated plasma (50 μl) for 1 min at 37 C prior to the addition of Thromborel S reagent (50 μl). The time taken for clot formations to occur (an upper maximal of 2 min was observed) were recorded using a Thrombotrak Solo coagulometer as per the manufacturer’s instructions. Water and sodium porcine mucosal heparin (203 IU/mg) were assayed as controls The EC_50_ values of all semi-synthetic, sulphated carbohydrates were determined using a sigmoidal dose response curved fitted post normalisation (with a 100% upper maximal at 2 mins; 0% lower maximal represented by the time required for the water control to clot normal human citrated plasma) with GraphPad Prism 6 software and compared to those obtained for the heparin control.

### Activated partial thromboplastin time (aPTT) coagulation assay

Serially diluted, sulphated carbohydrate samples (25 μl) were incubated with normal human citrated plasma (50 μl; NHSBT) and Pathromtin SL reagent (50 μl; Siemens) for 2 min at 37 C prior to the addition of calcium chloride (25 μl; 50 mM). The time taken for clot formations to occur (an upper maximal of 2 mins was observed) were recorded using a Thrombotrak Solo coagulometer (Axis-Shield) as per the manufacturer’s instructions. Water and sodium porcine mucosal heparin (203 IU/mg; VWR) were assayed as controls. The EC_50_ values of all semi-synthetic, sulphated carbohydrates were determined using a sigmoidal dose response curved fitted post normalisation (with a 100% upper maximal at 2 mins; 0% lower maximal represented by the time required for the water control to clot normal human citrated plasma) with GraphPad Prism 6 software and compared to those obtained for the heparin control.

### MTT cell proliferation assay

Potential toxic effects of sulphated carbohydrates on endothelial cells were screened against utilising the tetrazolium salt, 3–4,5 dimethylthiazol-2,5 diphenyl tetrazolium bromide (MTT) proliferation assay. Briefly, a serial dilution of the test carbohydrate was prepared and added to HUVEC cells (2×10^4^ cells) in a multiwell plate (Greiner); a positive control, the Golgi disruptor brefeldin A (10 μl, 10 ng.ml^-1^ in PBS), and PBS (10 μl, as a negative control) were also included. Post 48 hr incubation, MTT solution (10 μl, 0.5% w/v in PBS) was added to all wells for 4 hr at 37 C. Finally, the supernatants were discarded, the cells washed (PBS) and treated with dimethylsulfoxide (10 μl). Cell proliferation levels were ascertained indirectly by spectrophotometry at a λabs of 540 nm.

### Statistical analyses

**Fig 1:**
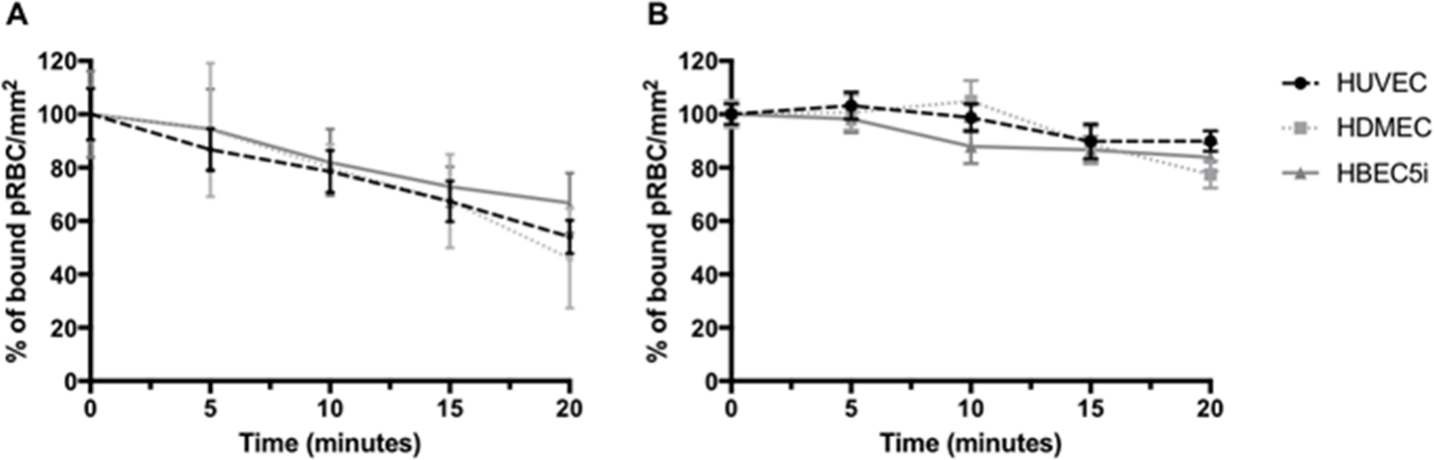
Reversal of ItG pRBC binding to different TNF-stimulated endothelial cells (HUVEC, HDMEC and HBEC5i) by 1 mg.ml^-1^ of (A) GSII and (B) PACS for 20 mins under flow conditions. pRBC bound were calculated every 5 mins and expressed as a percentage (%) bound pRBC.mm^-2^ compared to 0 min time point. Data for Fig 1 are provided in supporting information in S1 Data.

**Fig 2:**
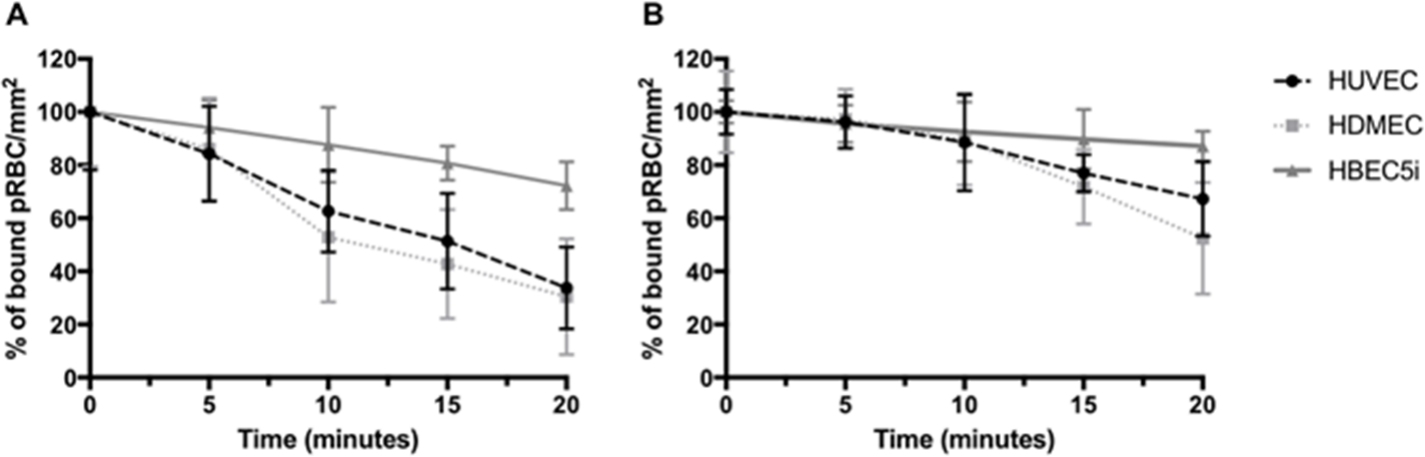
Reversal effect on A4 pRBC binding to different TNF-stimulated endothelial cells (HUVEC, HDMEC and HBEC) after flowing through 1mg.ml^-1^ of (**A**) GSII and (**B**) PACS for 20 mins under flow conditions. pRBC bound were calculated every 5 mins and expressed as a percentage (%) bound pRBC.mm^-2^ compared to 0 min time point. Data for Fig 2 are provided in supporting information in S2 Data.

**Fig 3:**
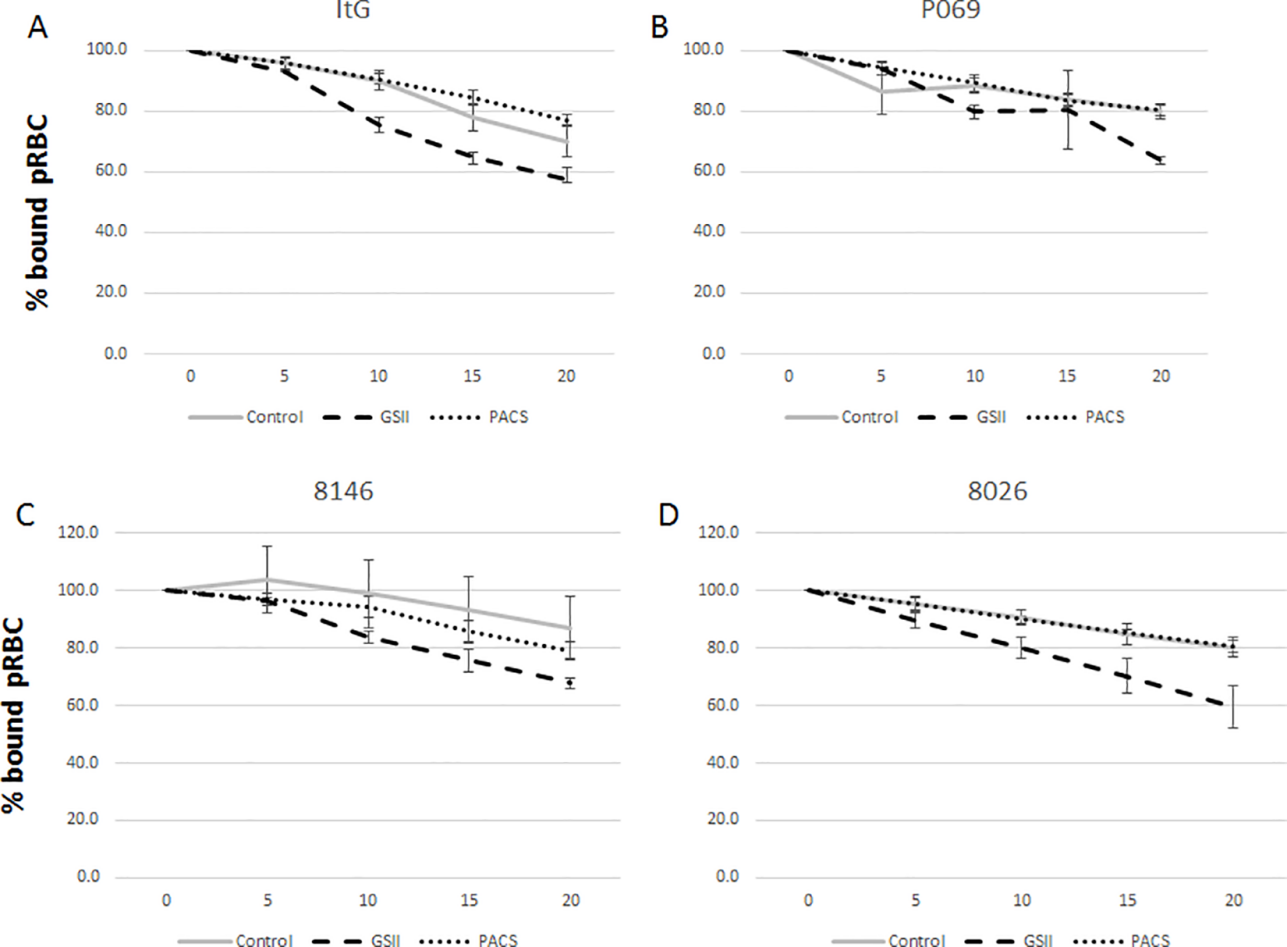
Reversal effect of 1 mg.ml^-1^ GSII and PACS on binding of lab-adapted patient isolates A) ItG; B) P069; C) 8146; D) 8026 to ICAM-1 under flow conditions. pRBC bound were calculated for every 5 mins and expressed as percentage (%) bound pRBC.mm^-2^ compared to 0 mins. Control is no compound (PBS only). X-axis is time in minutes after addition of GSII, PACS or PBS. Data for Fig 3 are provided in supporting information in S3 Data.

**Fig 4:**
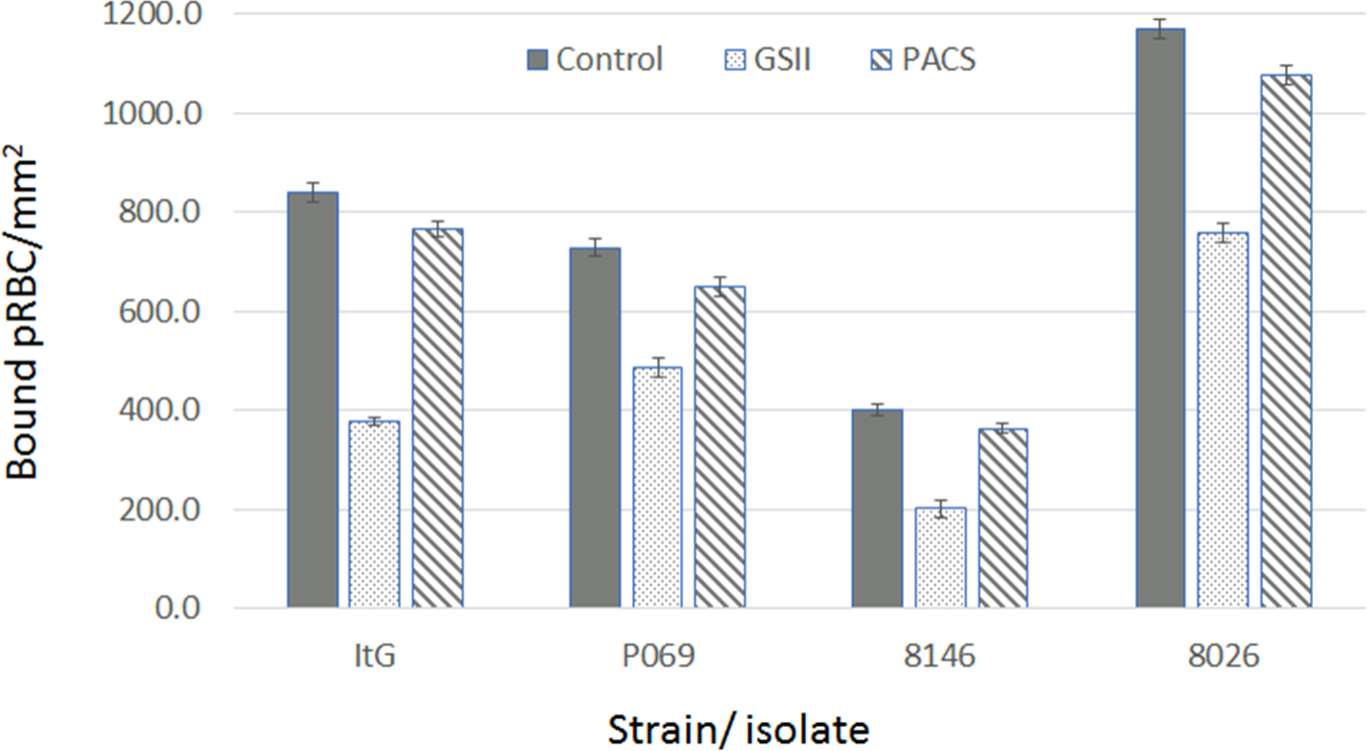
Reversal effect of 1 mg.ml^-1^ GSII and PACS on binding to HUVEC using lab-adapted patient isolates (P069, 8146 and 8026) under static assay conditions, with ItG used for comparison. The remaining bound pRBC were counted and expressed as bound pRBC.mm^-2^ mean ± standard deviation. Control is no compound (PBS only). Data for Fig 4 are provided in supporting information in S4 Data.

**Fig 5:**
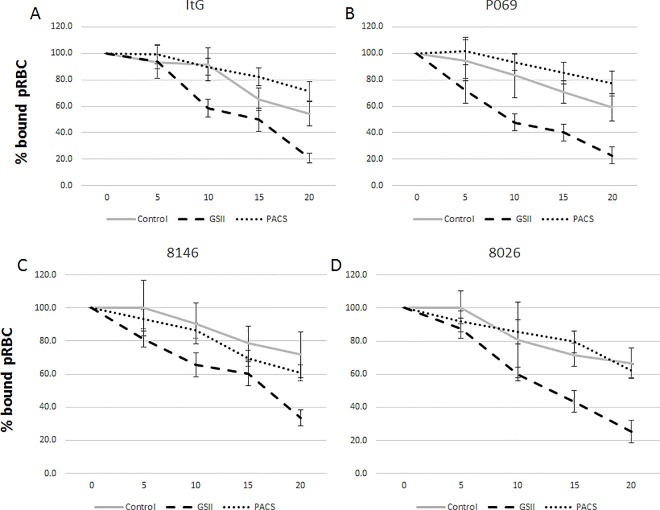
Testing 1 mg.ml^-1^ GSII and PACS for their ability to reverse existing binding under flow conditions. A) ItG; B) P069; C) 8146; D) 8026 reversal of binding on TNF-activated HUVEC. pRBC bound were determined every 5 minutes and expressed as percentage (%) bound pRBC. mm^-2^ compared to 0 mins. Control is no compound (PBS only). X-axis is time in minutes after addition of GSII, PACS or PBS. Data for Fig 5 are provided in supporting information in S5 Data.

Results shown are the mean of two independent experiments ± Standard Deviation (SD). A standard unpaired t-test was performed (Figs 1, 2 and 3), ANOVA (Kruskal Wallis with post-test) (Fig 4) and unpaired t-test (Fig 5) using GraphPad Instat3 software and considered significant when *P*<0.05.

Chemical characterisation methods for GSII are presented as supplementary information in S1 File.

## Results

Both static and flow based assays were used for this work, partly to allow comparison with other studies, which usually rely on static assays, but also to demonstrate the variation that can be observed in these two different formats.

### Inhibition of pRBC binding to endothelial cells by chemically modified, semi-synthetic anionic polysaccharides

A number of chemically modified, semi-synthetic anionic polysaccharides were screened for their anti-adhesion properties with a static based endothelial cell-binding assay against two well-characterised parasite lines, ItG and A4, for their binding to TNF-activated human primary endothelial cells HUVEC (human umbilical vein endothelial cells–large vessel endothelium) and HDMEC (human dernal microvascular endothelial cells). TNF activation was used to mimic the pro-inflammatory environment in a human host during malaria infection, and upregulates the expression of a number of cytoadherence receptors. One of the key differences between HUVEC and HDMEC, is that the latter expresses CD36, but HUVEC does not (or at very low levels that do not support CD36-based adhesion). Of the 44 compounds screened in a cell-based static assay (S1 Table; S1 Fig), 10 showed potential adhesion inhibition by producing 50% reduction in binding compared to the control receiving no treatment (Table 1). From the flow-based screening we identified only two compounds:glycogen type 2 sulfate from Oyster (MS34, GSII) and phenoxyacetylcellulose sulfate (MS40, PACS)) that showed a significant adhesion inhibitory effect; they reduced the binding of the A4 strain up to 70% and achieved 10–40% reduction in binding of the ItG strain to TNF activated HDMEC in comparison to the un-treated control (S2 Fig).

**Table 1:** Compounds identified in primary screen.

Compound	Description	Carbohydrate composition
**1**	Glycogen sulfate (type II) (GS)	α(1→4), α(1→6), polyglucan
**2**	Phenoxyacetyl cellulose sulfate (PACS)	β(1→4) phenoxyacetylated polyglucan
**3**	Ethyl cellulose sulfate	β(1→4) acetylated polyglucan
**4**	Gum Arabic sulfate	β(1→3), β(1→3) polygalactan core
**5**	Starch sulfate	α(1→4) polyglucan
**6**	Poly-D-methylgalacturonic acid sulfate	α(1→4) methylated polygalacturonic acid
**7**	Poly-D-galacturonic acid sulfate	α(1→4) polygalacturonic acid
**8**	Tragacanth sulfate	α(1→4) polygalacturonic acid, β(1→3) xylan
**9**	Hydroxypropyl methyl cellulose sulfate	β(1→4) hydroxpropylated polyglucan
**10**	Paramylon sulfate	β(1→3) polyglucan

### Reversal effect of chemically modified, semi-synthetic anionic polysaccharides on endothelial cells

Having identified GSII and PACS as capable of inhibiting parasite adhesion, we further screened these compounds in terms of their ability to reverse the binding of two-laboratory parasite strains ItG (Fig 1) and A4 (Fig 2) to distinct endothelial cell lines (HUVEC, HDMEC and HBEC5i) using a cell-based flow adhesion assay. HBEC5i is an immortalised human brain microvascular cell line with a similar profile of receptor expression to HUVEC. For this secondary screen, the comparisons for inhibitory effects were made to the situation at 0 min, but it is known that there is a low-level loss of binding of PRBC during flow adhesion assays, and this was incorporated into later experiments by running a ‘no compound/ PBS’ control for the same period. GSII disrupted binding with 40% to 60% reduction of pRBC on all these cell types, comparing 0 min with 20 min exposure to the compounds (Figs 1A and 2A), whereas PACS showed smaller, variably significant reversal effects across all the endothelial cells (Figs 1B and 2B) (Table 2).

**Table 2:** p-values for cytoadherence assays: (unpaired t-test: Figs 1 and 2). Comparison is between t = 20 min and t = 0 min and not a ‘no compound’ control at 20 min.

	ItG		A4	
	GSII	PACS	GSII	PACS
HUVEC	0.0079	0.0611	0.0037	0.018
HDMEC	0.0128	0.2326	0.0053	0.024
HBEC5i	0.002	0.0668	0.0098	0.0661

### Reversal effect of GSII and PACS on other parasite isolates

To examine the effect of both GSII and PACS using a broader range of parasite variants, three recently laboratory-adapted, ICAM-1-selected patient isolates (P069, 8146 and 8026) were used in addition to ItG [23]. Initially the ability of the compounds to disrupt pRBC already bound to ICAM-1 protein under flow conditions was tested. The reversal was similar for each parasite isolate, albeit with some variation, and around 20–30% pRBC were removed with GSII being more effective than PACS (Fig 3 and Table 3), but only P069/ GSII was statistically significant. The reduction in binding was significant when using TNF activated HUVEC cells with a 40–50% reduction under static conditions that was significant with GSII (Fig 4 and Table 4), but lower and variably significant with PACS. Under flow conditions, GSII showed consistent reductions in binding of all isolates tested again reaching significant or near significant levels, unlike PACS, which showed no significant reductions in binding when compared to ‘no-compound’ control (Fig 5 and Table 5).

**Table 3:** p-values for cytoadherence assays: (unpaired t-test: Fig 3). Comparison is between control (no compound) at 20 min with the relevant compound treatment at 20 min.

	ItG	P069	8146	8026
GSII	0.1094	0.0134	0.1328	0.0702
PACS	0.224	0.7762	0.4295	0.9271

**Table 4:** p-values for cytoadherence assays: (ANOVA-Kruskal-Wallis: Fig 4). Comparison is with the relevant control for each isolate.

	ItG	P069	8146	8026
GSII	< 0.001	< 0.01	< 0.001	< 0.001
PACS	< 0.05	> 0.05	> 0.05	< 0.01

**Table 5:** p-values for cytoadherence assays: (unpaired t-test: Fig 5). Comparison is between control (no compound) at 20 min with the relevant compound treatment at 20 min.

	ItG	P069	8146	8026
GSII	0.0418	0.0522	0.0673	0.0361
PACS	0.171	0.2095	0.407	0.5997

### Anticoagulant potential of GSII

**Fig 6:**
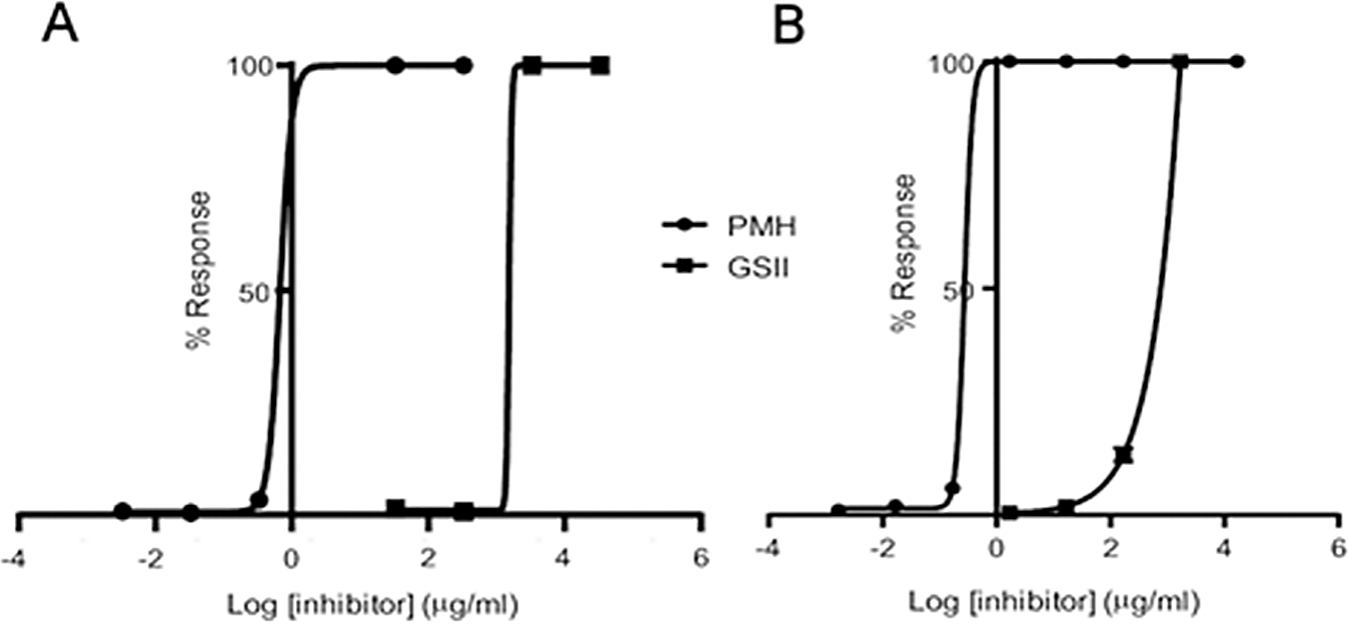
A) Prothrombin time (PT) assay to determine EC_50_ for the compounds for coagulation. 100% represents the inhibition of clotting, determined as a PT of >120 seconds, 0% represents a normal PT clotting time for pooled human plasma (≈ 13–14 seconds). EC_50_ Porcine Mucosal Heparin (PMH, the clinically used antihrombotic agent) = 2.74 x 10^−1^ μg.mL^-1^; EC_50_ GSII = 5.44 x 10^4^μg.mL^-1^. B) Activated partial thromboplastin time (aPTT) assay. 100% represents the inhibition of clotting, determined as a aPTT of >120 seconds, 0% represents a normal PT clotting time for pooled human plasma (≈ 35 seconds). EC_50_ Porcine Mucosal Heparin (PMH) = 6.74 x 10^−1^ μg.mL^-1^; EC_50_ PACS = 1.53 x 10^4^ μg.mL^-1^. Data for Fig 6 are provided in supporting information in S6 Data.

In light of the reversal efficacy of GSII, the ability of this sulfated carbohydrate to perturb coagulation within pooled human plasma was determined. The prothrombin time (PT) and activated partial thromboplastin time (aPTT) were measured for GSII, thereby determining the overall effect on the extrinsic and intrinsic coagulation pathways respectively (both assays also include the common coagulation pathway). The sulfated carbohydrate porcine mucosal heparin (sodium), an approved clinical anticoagulant of known activity (201 IU.mg^-1^) was employed for comparison as a relevant control. The anticoagulant potential of GSII is highly attenuated when compared to that of the sodium heparin control, in both the PT (EC_50_ of 5.44 x 10^4^ μg.mL^-1^ versusEC_50_ of 2.7 x 10^−1^, respectively; Fig 6A) and aPTT (EC_50_ of 1.53 x 10^4^ versus 6.74 x 10^−1^ μg.mL^-1^, respectively; Fig 6B) coagulation assays.

### Potential cytotoxicity of GSII

**Fig 7:**
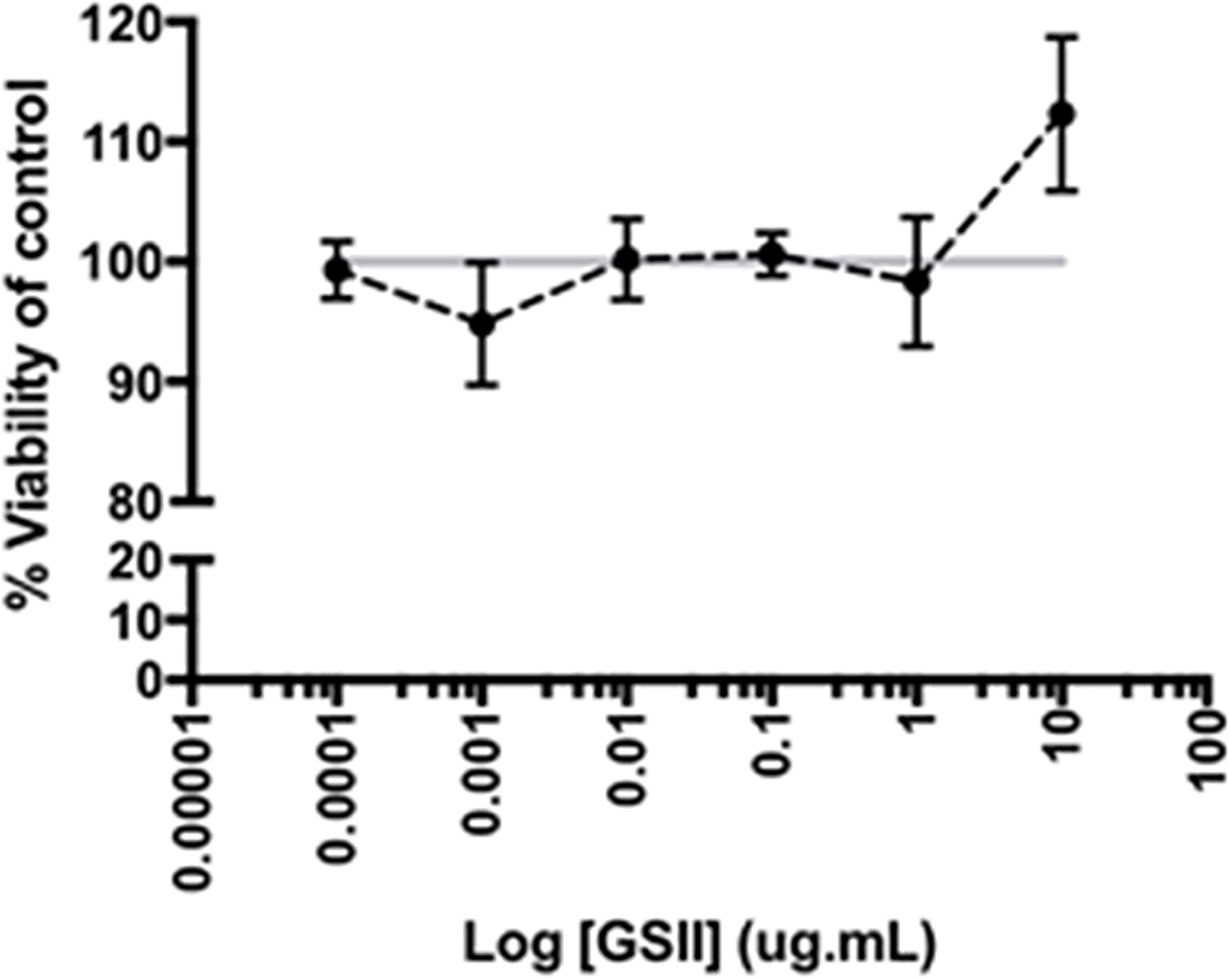
Cell viability measured indirectly using the MTT cell proliferation assay after incubation for 48 hours with varying concentrations of GSII on HUVEC cells. All results are expressed as a percentage relative to a non-toxic control (PBS). Brefeldin A (a known Golgi disruptor) at 10 ng.ml^-1^ was used as a toxicity control (data not shown). The data plotted is the mean ±S.D. of triplicate values. Statistical analysis was performed using an unpaired t-test. All concentrations of GSII assayed were statistically insignificant from the PBS control (i.e. *P* > 0.05). Data for Fig 7 are provided in supporting information in S7 Data.

Potential cytotoxic effects of GSII were screened for using the widely adopted MTT assay, which detects the chromogenic change that occurs upon the mitochondrial reduction of the tetrazol dye MTT to yield formazan in living cells. This reduction does not occur in deceased cells, thereby acting as an indirect measure of toxicity, through the reduced levels of cell proliferation that would be observed when a cytotoxic agent is present, compared to that of the normally proliferating cell population. The incubation of HUVEC endothelial cells in the presence of GSII at increasing concentrations up to 10 mg.mL^-1^ showed no apparent evidence of cytotoxicity when compared to the PBS control (Fig 7).

## Discussion

Erythrocytes infected with *P*. *falciparum* can bind to endothelial receptors, leading, in part, to the clinical manifestations associated with SM. Consequently, molecules that can inhibit or interrupt these interactions may have a role in improving understanding of host-parasite biology, as well as in developing new therapies for severe disease. Highly sulfated polysaccharides not only inhibit binding of pRBC to CSA [12–14], but can also reverse pRBC adhesion in the placenta during pregnancy [25, 26]. Interactions between pRBC and host cell membranes involve multiple interactions between several distinct ligands and receptors. The cell surface receptors, including ICAM-1, CD36, EPCR, complement receptor 1 and chondroitin sulfate-A, bind to different regions of the PfEMP-1 protein (the cysteine rich inter-domain region (CIDR) and Duffy binding-like domains (DBL) [27, 28]. The extent of involvement of these receptors varies between parasite variants and may correspond to pathology [29]. For example, chondroitin sulfate A (repeating -4 GlcA β(1–3) GalNAc4S β(1- disaccharide units) is implicated in placental malaria [25].

The interactions between pRBCs and host cells almost certainly involve manifold protein-carbohydrate contacts across the interacting surfaces, in addition to HS. These lead to strong interactions between cells that can be much more difficult to dislodge than the simple sum of the individual components. This multi- or polyvalent effect is why small molecules are unable to reverse binding interactions at reasonable concentrations. In contrast, if larger molecules are employed, then effective inhibition becomes possible as long as the appropriate geometric and stereochemical requirements to make effective bonds with receptors are present [30].

Receptor-ligand binding is a dynamic process and this is especially relevant in the case of multivalent interactions. In cell-to-cell interactions, multivalency exists at two distinct levels. In the first, there is multivalency across the surface of the interacting cells, formed by many individual molecular contacts. In the second, at the more detailed molecular level, there are multiple bonds formed between individual interacting molecules, e.g. carbohydrate-protein or protein-protein, which could comprise, for example, hydrogen bonds at several locations. The dynamic aspects of both situations are essentially similar in nature, while being very different in scale. Polyvalent interactions can be viewed as a series of discrete interactions, spread out in space, but dynamic in nature and in a constant state of change. Each interacting pair spends some time bound and some unbound, but this lacks overall synchronisation. A small molecule inhibitor can only bind one, or a few, of the available binding sites as and when they become available, but cannot achieve efficient competition because, without resorting to extremely high concentrations, it can never occupy sufficient sites locally to dislodge the original binding molecules. If, on the other hand, a larger inhibitor with the appropriate spatial and stereochemical characteristics to enable it to make first one, two and then several interactions is introduced, then this can effectively compete with the ligand-receptor system and eventually dislodge the original binding partners to replace one of them with itself. Such effective competition can only be achieved by larger molecules, including polysaccharides.

The inhibitory interactions described here are not simply charge-driven. There is an element of complementarity, supported by studies showing that low-sulfated structures can inhibit pRBC binding [16]. GSII must possess specific dimensions and stereochemical characteristics that enable it to act as an effective multivalent inhibitor at the level of both molecular and cellular interactions. This requirement for particular geometric and charge distribution is seen in that simply increasing charge does not necessarily improve inhibition [see compound 36, supplementary data]. Additionally, polysaccharide inhibitors are not necessarily acting only via a single type of ligand–receptor interaction, so polysaccharide heterogeneity may be an advantage.

Ten potential inhibitory polysaccharides were identified (Table 1) from a library of 44 modified semi-synthetic anionic polysaccharides using a simple static binding assay. These exhibited significant inhibition with two lab-adapted *P*. *falciparum* lines, A4 and ItG under static conditions (S1 Fig). We then used the more complex, but physiologically relevant, flow-based binding assay to investigate the ability of the ten polysaccharides to inhibit binding of these two laboratory-adapted strains. GSII and PACS gave approximately 60–70% reduction in binding when using A4, but not ItG (S2 Fig). The interaction between pRBC and endothelial cells varies with the composition of the variant surface protein expressed on the different pRBC lines and the repertoire of endothelial receptors on the host cells. Comparing binding efficiencies is complicated by the different assay platforms used, however, under static conditions ItG supports higher binding to ICAM-1 and CD36 than A4, which is also seen under static conditions for HUVEC and HDMEC [22]. Under flow conditions, binding to endothelial cells for these two parasite lines is different; A4 showing higher binding to TNF-induced HUVEC and both lines showing essentially equivalent levels of binding to HDMEC. Therefore, the variability seen in the level of inhibition between the different parasite lines may be due to differential inhibition of a range of interactions, and no preference for any specific host receptor can be discerned. This is un-surprising since the polysaccharide library was not designed to target specific host-parasite interactions for cytoadherence.

Polysaccharides such as dextran sulfate and fucoidan can inhibit adhesion of *P*. *falciparum* to host receptors such as CSA and CD36 [12–14] and regioselectively modified polysaccharides, including modified carrageenans inhibit binding of pRBC to CD36 [31, 32], as well as modified heparin structures [18]. GSII can now be added to this list but, the greater challenge related to whether GSII or PACS could reverse bound pRBC, which is the situation found clinically in SM. To answer this, three different EC (HUVEC, HDMEC and HBEC5i) were used in combination with A4 and ItG to provide a further screen for activity prior to more detailed analysis. Brain EC is potentially important as it may indicate whether GSII and PACS could reduce sequestration in this tissue, as detected in cerebral malaria.

GSII gave a better response than PACS on different EC (HUVEC, HDMEC and HBEC5i) and was more effective in reversal of A4 binding to HUVEC and HDMEC cells compared to human brain microvascular endothelial cells (HBMEC). PACS did not show any ability to reverse binding with ItG but gave slightly better results on reversing A4 binding to HDMEC and HUVEC. Although GSII and PACS showed different effects on reversing adhesion of both lab-adapted strains, neither had a significant effect on HBEC5i, which is a concern in terms of developing either compound as an adjunct therapy for CM. However, the HBEC5i used here is an immortalized rather than a primary cell line and this may have influenced the level of binding and inhibition. Differential binding of parasite lines to HBEC5i and primary HBMEC has been reported [33]. Further work on primary brain endothelium and tissue sections will be needed, as will understanding the impact of releasing many rigid pRBC into the circulation on the health of the patient.

GSII and PACS were further investigated by testing a panel of recently laboratory-adapted parasite isolates (8146, 8026 and P069). Isolate 8146 is a strong ICAM-1 binder, with 8206 and P069 showing slightly lower levels of adhesion to this receptor [23]. Each parasite strain showed roughly equivalent reversal responses under static and flow conditions, with up to 80% reduction under flow (Figs 4 and 5) suggesting that GSII could have broad application in terms of pRBC binding inhibition. GSII contains low sulfate (and charge) and the results support the work of McCormick *et al*., who showed that low sulfated glycoconjugates were able to modulate binding of pRBC to different receptors [16].

Significant anticoagulant activity is a well-known off-target effect that has previously hampered the application of some, but not all, sulphated molecules as potential therapeutic agents. The PT and aPTT coagulation times of GSII suggest that it possesses negligible anticoagulant potency (10^−6^) compared to pharmaceutical heparin. Furthermore, GSII does not significantly perturb endothelial cell proliferation by tetrazolium based dye, MTT, suggesting that it possesses favourable bioactivity, is minimally anticoagulant and non-toxic.

This work has demonstrated the potential of chemically modified, semi-synthetic anionic polysaccharides to both inhibit and reverse cytoadherence in malaria and offers potential for the future development of pharmaceutical agents based on these materials.

## Supporting information

S1 Fig Screening compounds under static assay conditions. Binding response of ItG (top) and A4 (bottom) to modified polysaccharide compound at 1 mg/ml on TNF-stimulated HUVEC and HDMEC under static condition (single screening). pRBC binding (3%, parasitemia; 1% HCT) observed after polysaccharide treatment for one hour. The remaining bound pRBC after gravity wash were counted and expressed as % bound pRBC/ mm^2^ (N = 1) compared to control, without polysaccharide.
(TIF)

S2 Fig Screening compounds under flow assay conditions. Binding response of ItG and A4 to modified polysaccharides compounds at 1 mg/ml on TNF-stimulated HDMEC under flow conditions. The remaining bound parasites after 20 mins wash was counted and expressed as % bound pRBC/ mm^2^ ± standard deviation compared to control without polysaccharide. ND; not done. MS34 (ItG and A4) & MS40 (A4), *P* < 0.05 (compared to control).
(TIF)

S3 Fig Characterisation of GSII. ^1^H NMR spectrum of GSII at 400 MHz, with 128 scans, 2s delay. Inset—Comparison of ^1^H NMR spectra of GSII and its unsulfated precursor, glycogen type II. This is provided as background information and is not cited in the paper.
(TIF)

S1 Table Sulfated carbohydrates assayed. A list of all the compounds screened in this paper.
(DOCX)

S1 File Supplementary methods. Description of methods associated with chemical characterisation of GSII.
(DOCX)

S1 Data Primary data supporting Fig 1. (XLSX)

S2 Data Primary data supporting Fig 2. (XLSX)

S3 Data Primary data supporting Fig 3. (XLSX)

S4 Data Primary data supporting Fig 4. (XLSX)

S5 Data Primary data supporting Fig 5. (XLSX)

S6 Data Primary data supporting Fig 6. (XLSX)

S7 Data Primary data supporting Fig 7. (XLSX)
