# Supplementary figures and images for: A semi-synthetic glycosaminoglycan analogue inhibits and reverses Plasmodium falciparum cytoadherence
Source: PLoS One. 2017 Oct 18;12(10):e0186276. doi: 10.1371/journal.pone.0186276 (PMC5646806; doi:10.1371/journal.pone.0186276)

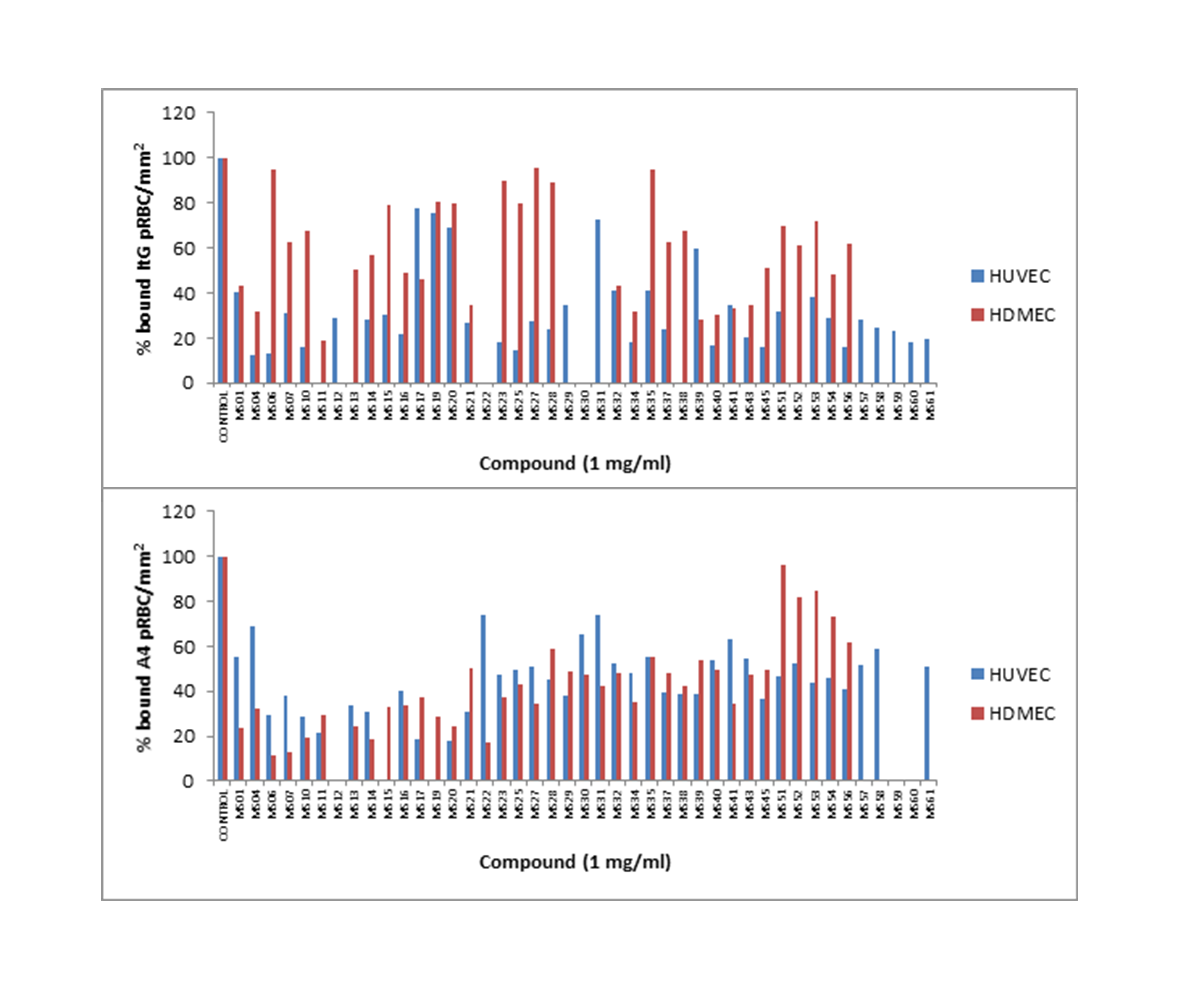

Supplement: S1 Fig — Binding response of ItG (top) and A4 (bottom) to modified polysaccharide compound at 1 mg/ml on TNF-stimulated HUVEC and HDMEC under static condition (single screening). pRBC binding (3%, parasitemia; 1% HCT) observed after polysaccharide treatment for one hour. The remaining bound pRBC after gravity wash were counted and expressed as % bound pRBC/ mm2 (N = 1) compared to control, without polysaccharide. (TIF) [file pone.0186276.s001.tif]

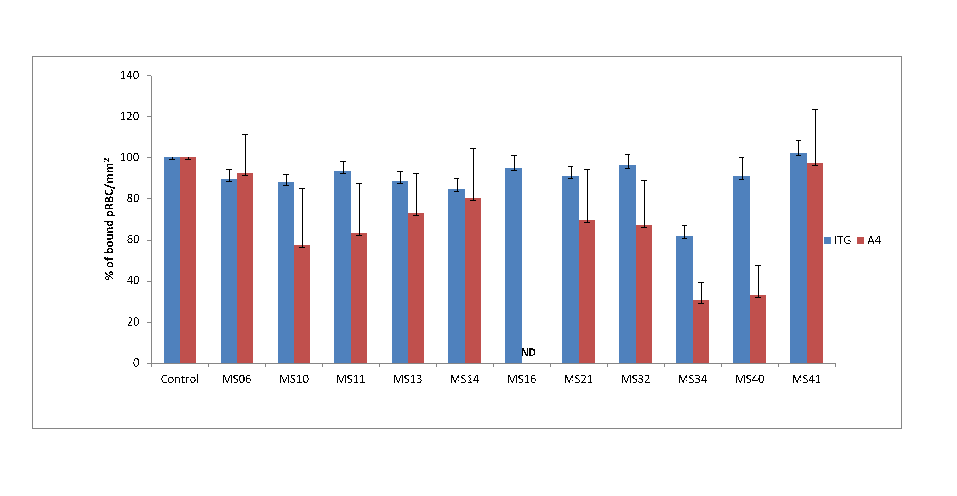

Supplement: S2 Fig — Binding response of ItG and A4 to modified polysaccharides compounds at 1 mg/ml on TNF-stimulated HDMEC under flow conditions. The remaining bound parasites after 20 mins wash was counted and expressed as % bound pRBC/ mm2 ± standard deviation compared to control without polysaccharide. ND; not done. MS34 (ItG and A4) & MS40 (A4), P < 0.05 (compared to control). (TIF) [file pone.0186276.s002.tif]

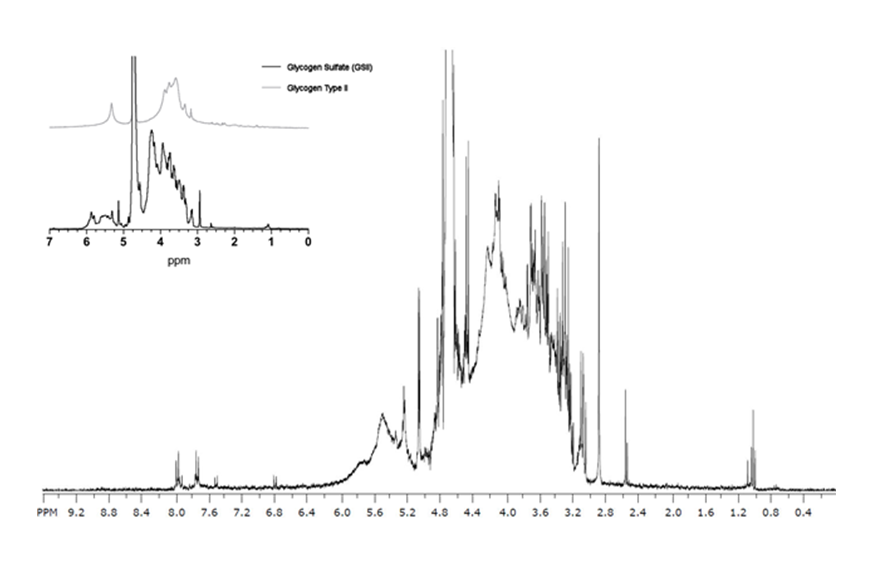

Supplement: S3 Fig — 1H NMR spectrum of GSII at 400 MHz, with 128 scans, 2s delay. Inset—Comparison of 1H NMR spectra of GSII and its unsulfated precursor, glycogen type II. This is provided as background information and is not cited in the paper. (TIF) [file pone.0186276.s003.tif]
