# Supplementary material for: A semi-synthetic glycosaminoglycan analogue inhibits and reverses Plasmodium falciparum cytoadherence
Source: PLoS One. 2017 Oct 18;12(10):e0186276. doi: 10.1371/journal.pone.0186276 (PMC5646806; doi:10.1371/journal.pone.0186276)
Supplement: S1 Table — A list of all the compounds screened in this paper. (DOCX) [file pone.0186276.s004.docx]

**Supplementary Table 1: Sulfated carbohydrates assayed**

Compound # MS# Compound name Compound # MS# Compound name

1 MS34 Glycogen sulfate (type II) (GS)

2 MS40 Phenoxyacetyl cellulose sulfate (PACS)

3 MS04 Ethyl cellulose sulfate

4 MS10 Gum Arabic sulfate

5 MS13 Starch sulfate

6 MS14 Poly-D-methylgalacturonic acid sulfate

7 MS33 Poly-D-galacturonic acid sulfate

8 MS21 Tragacanth sulfate

9 MS32 Hydroxypropyl methyl cellulose sulfate

10 MS41 Paramylon sulfate

11 MS01 Methyl 2-hydroxyethyl cellulose sulfate

12 MS06 Gellan Gum sulfate

13 MS07 Alginic acid sulfate

14 MS45 Laminarin sulfate

15 MS51 Fucogalactan sulfate

16 MS11 Gum Rosin sulfate

17 MS12 Styrax sulfate

18 MS25 Dextrin sulfate

19 MS15 Gum Mastic sulfate

20 MS16 Potassium Pectate sulfate

21 MS17 Inulin sulfate

22 MS43 Stachyose sulfate

23 MS19 Carboxymethyl cellulose sulfate

24 MS20 i-carrageenan persulfate

25 MS53 l- carrageenan persulfate

26 MS52 k-carrageenan persulfate

27 MS22 Amylopectin sulfate

28 MS23 Karaya Gum sulfate

29 MS54 Konjac glucomannan sulfate

30 MS56 Psyllium seed gum sulfate

31 MS57 Scleroglucan sulfate

32 MS27 Agarose sulfate

33 MS28 Xylan sulfate

34 MS29 Arabic acid sulfate

35 MS30 Amylose sulfate

36 MS31 Dextran sulfate

37 MS39 b-1,3 glucan sulfate

38 MS35 Methyl cellulose sulfate

39 MS58 Welan sulfate

40 MS37 Pullulan sulfate

41 MS38 Levan sulfate

42 MS59 Sodium alginate sulfate

43 MS60 Polyproyleneglycol alginate sulfate

44 MS61 Tamarind gum sulfate
