# Supplementary material for: A semi-synthetic glycosaminoglycan analogue inhibits and reverses Plasmodium falciparum cytoadherence
Source: PLoS One. 2017 Oct 18;12(10):e0186276. doi: 10.1371/journal.pone.0186276 (PMC5646806; doi:10.1371/journal.pone.0186276)
Supplement: S1 File — Description of methods associated with chemical characterisation of GSII. (DOCX) [file pone.0186276.s005.docx]

**Supplementary Methods**

**Characterisation of the sulfate level.**

The overall sulfation level of GSII was 15.4 mg of sulfate per g of GSII as determined by the method of Terho and Hartiala, (Anal. Biochem. 1971, 41(2):471-6). Briefly, dextran sulfate of a known degree of sulfation was hydrolysed in 1 M hydrochloric acid at 100°C for 2 hours and lyophilised. The dry product was reconstituted in HPLC water (1 mg.ml^-1^) and serial dilutions performed (to establish a calibration curve) prior to the addition of 100 mM acetic acid, 50 uM barium chloride, 800 μM sodium bicarbonate, 0.14 mM rhodizonic acid and 3.4 mM L(+)-ascorbic acid. The solution was incubated in darkness for 10 mins at room temperature to permit colour development. The absorbance of the solution was ascertained at λ_abs_= 520 nm. The assay was repeated for GSII and the mass of sulfate per gram of polysaccharide calculated from the dextran sulfate calibrant.

**Characterisation of the molecular weight and polydispersity.**

The Mw, Mn and the degree of polydispersity of GSII were determined by size exclusion chromatography (SEC) using Superdex 100 and Superdex 500 columns (10 mm x 500 mm) in series with a running buffer of 250 mM ammonium chloride held isocratic at 200 μL.min^-1^. The M_w_ and M_n_ of GSII were determined as 8.1 kDa and 3.9 kDa respectively, with a polydispersity index (PDI) of 2.08 when using commercially sourced size exclusion calibrants (Biorad #151-1901).
